# Supplementary material for: Changes in Sexual Behavior and Satisfaction and Violent Behavior during COVID-19 Lockdown: Explorative Results from the Italian Cross-Sectional Study of the I-SHARE Multi-Country Project
Source: Int J Environ Res Public Health. 2024 Jan 15;21(1):96. doi: 10.3390/ijerph21010096 (PMC10815040; doi:10.3390/ijerph21010096)
Supplement: Supplementary file 1 [file ijerph-21-00096-s001.zip › ijerph-2795872-supplementary.pdf]

I-SHARE: International Sexual and Reproductive Health Survey in the time of COVID-19

CONSENSO INFORMATO

Gentilissimo/a,

La invitiamo a partecipare a questa ricerca sulla salute sessuale e riproduttiva e sul benessere durante il momento di crisi dovuto al COVID-19: lo studio I-SHARE.
L'obiettivo di questo studio è quello di investigare come le misure di confinamento sociale (lockdown, stare a casa, distanziamento sociale, limitazione alle uscite essenziali, ecc…) prese dal Governo del Suo paese durante la crisi sanitaria del COVID-19 hanno influito sulla sua situazione familiare, le sue relazioni e l'accesso ai servizi della salute sessuale e riproduttiva (come la contraccezione e servizi di assistenza prenatale). Pertanto, lo studio contiene varie domande nelle quali Le verrà chiesto di comparare la Sua situazione prima e dopo l'introduzione delle misure di distanziamento sociale da COVID-19. Lo studio è organizzato dal Dipartimento di Psicologia Dinamica e Clinica dell'Università degli studi di Roma "Sapienza" in collaborazione con istituzioni accademiche di altri trenta paesi (https://ishare.web.unc.edu/)

La partecipazione a questo studio è importante e ci permetterà di ottenere una panoramica completa su come la crisi dovuta al COVID-19 ha influito sulla vita delle persone. La partecipazione richiederà circa 15 minuti.

La partecipazione allo studio è anonima e volontaria. Può interrompere la compilazione in qualsiasi momento.
Ulteriori informazioni riguardanti la ricerca, come verranno processati i dati e come proteggeremo la Sua privacy possono essere trovate nel seguente link. Deve avere più di 18 anni per partecipare allo studio.

LINK: https://forms.gle/RC2HESXAnA7TkxXM7

La ringraziamo in anticipo per il Suo tempo e la Sua partecipazione.

Cordialmente,

Con la presente dichiaro che, come partecipante allo studio “I-SHARE: International Sexual and Reproductive Health Survey in the time of COVID-19”:

- Ho letto e compreso la lettera informativa per i partecipanti. Sono stato informato sulla natura, sulla durata e sullo scopo dello studio.
- Mi è stata offerta la possibilità di avere informazioni aggiuntive.
- Ho compreso che la partecipazione allo studio è di carattere volontario. So di poter ritirare la mia partecipazione in ogni momento senza doverne giustificare il motivo.
- Sono consapevole che questo studio è stato approvato dal Comitato etico del Dipartimento di Psicologia Dinamica e Clinica dell’Università degli studi di Roma “Sapienza” per la raccolta nazionale di dati, e che questo studio verrà condotto in accordo con le linee guida per una buona pratica clinica (ICH/GCO) e la dichiarazione di Helsinki, progettata per tutelare i partecipanti coinvolti nelle ricerche. Sono a conoscenza del fatto che la partnership I-SHARE otterrà l’autorizzazione da parte dei paesi partecipanti, al fine di acquisire dati anonimi per confrontarli con quelli di paesi diversi. Questo processo verrà formalizzato tramite accordi di condivisione dei dati e attraverso l’approvazione del comitato etico per supportare l’analisi secondarie dei dati ottenuti dalle rispettive istituzioni. La decisione di partecipare non è, in nessun modo, il frutto di un impeto momentaneo.
- Autorizzo i ricercatori a immagazzinare i miei dati in via confidenziale in accordo con la politica di sicurezza della gestione dei dati del Dipartimento di Psicologia Dinamica e Clinica dell’Università degli studi di Roma “Sapienza” in modo che questi dati possano essere riutilizzati in un contesto formativo e futura ricerca scientifica.
- Autorizzo i ricercatori a elaborare e riportare i miei risultati in via riservata.
- Sono a conoscenza di poter contattare il responsabile della protezione dei dati del Dipartimento di Psicologia Dinamica e Clinica dell’Università degli studi di Roma “Sapienza” (filippo.nimbi@uniroma1.it; stefano.eleuteri@uniroma1.it),per ulteriori informazioni riguardo la protezione dei miei dati.
- Una volta compilato il questionario esso diventerà totalmente riservato, in seguito non avrò la possibilità di cambiare, modificare o cancellare le mie risposte.

Dichiaro di aver compiuto 18 anni

- Si
- No

Spuntando questa casella, sono d’accordo con quanto sopra dichiarato, do il mio consenso e desidero partecipare allo studio.

Lettera informativa per i partecipanti dell'SRH COVID-19 Study

Sotto può trovare ulteriori informazioni riguardo lo studio "I- SHARE, Sexual and Reproductive Health and wellbeing in times of COVID-19". Se dovesse avere domande dopo la lettura di questo documento, i ricercatori saranno felici di risponderle. Può trovare i contatti in fondo a questa pagina. Per favore, chiuda questa finestra dopo averne letto il contenuto per continuare con la compilazione del questionario. Può anche scaricare questo documento per avere la possibilità di leggere l'informativa anche in seguito.

Perché studiamo la salute sessuale e riproduttiva durante il periodo del COVID-19?

Questo studio vuole contribuire ad una migliore comprensione dell'impatto delle misure di confinamento (stare a casa, distanziamento sociale, uscite essenziali, ecc…) prese dal Governo del Suo paese durante la crisi sanitaria dovuta al COVID-19 sulla salute sessuale e riproduttiva e sul benessere. I risultati di questo studio contribuiranno quindi allo sviluppo di raccomandazioni che potrebbero limitare il più possibile le conseguenze negative di queste misure.

Chi conduce questa ricerca?

Il Dipartimento di Psicologia Dinamica e Clinica dell'Università degli studi di Roma "Sapienza" è incaricato di portare avanti questo studio guidato dal Dott. Filippo Maria Nimbi e dal Dott. Stefano Eleuteri.
Questo studio è stato condotto egualmente in altri vari paesi del mondo (https://ishare.web.unc.edu/). La partnership I-SHARE otterrà l'autorizzazione da parte dei paesi partecipanti, al fine di acquisire dati anonimi per confrontarli con quelli di paesi diversi. Questo processo sarà formalizzato in accordi di condivisione dei dati e attraverso l'approvazione del comitato etico per sostenere l'analisi secondaria dei dati ottenuti dalle rispettive istituzioni.

Che garanzie ci sono che la ricerca verrà svolta correttamente?

Questo studio è stato approvato dal comitato etico del Dipartimento di Psicologia Dinamica e Clinica dell'Università degli studi di Roma "Sapienza". Questo studio sarà condotto in conformità con le linee guida per una buona pratica clinica (ICH/GCP) e con la dichiarazione di Helsinki.
La Sua partecipazione a questo studio è totalmente riservata. I ricercatori non conosceranno il Suo nome, o il suo indirizzo.
I ricercatori aderiscono alle disposizioni della legislazione sulla protezione dei dati.

Chi può partecipare a questo studio?
Qualsiasi persona che vive in Italia e che abbia compiuto 18 anni può partecipare a questo studio.

Sono obbligato a partecipare?
La Sua partecipazione a questo studio è interamente volontaria. La non-partecipazione non Le causerà alcun danno. Può ritirare il suo consenso in ogni momento.

Cosa succederà con i miei dati?
Le Sue risposte verranno analizzate in forma aggregata. Tutte le risposte verranno elaborate in documenti, relazioni o pubblicazioni (in giornali scientifici o conferenze) riguardo lo studio, nei quali non potrà essere identificata nessuna risposta individuale. La Sua partecipazione a questo studio è interamente riservata. Garantire la riservatezza, tuttavia, implica che non sarà possibile cambiare, rivedere o cancellare i dati in seguito.
I dati della ricerca saranno usati solamente per scopi scientifici, educazionali o statistici. Al fine di facilitare il confronto tra i vari paesi, i dati della ricerca possono essere condivisi con i ricercatori, che a loro volta saranno tenuti a rispettare la legislazione applicabile in materia di protezione dei dati che implementano l'I-SHARE Study nei loro rispettivi paesi.

A livello nazionale, i set di dati saranno conservati presso l'Istituto di Sessuologia Clinica, Via Savoia 78, Roma. La partnership I-SHARE otterrà l'autorizzazione da parte dei paesi partecipanti, al fine di acquisire dati anonimi per confrontarli con quelli di paesi diversi. Questo processo sarà formalizzato in accordi di condivisione dei dati e attraverso l'approvazione del comitato etico per sostenere l'analisi secondaria dei dati ottenuti dalle rispettive istituzioni.

Il Dott. Filippo Maria Nimbi e il Dott. Stefano Eleuteri sono responsabili del trattamento nazionale dei dati.

Lei ha il diritto di presentare un reclamo sulla gestione dei dati a:

- Livello nazionale: Responsabile della protezione dei dati (RPD) è il Dott. Andrea Bonomolo responsabileprotezionedati@uniroma1.it

Cosa succede in caso di danni o lesioni a seguito della partecipazione allo studio?
Nel caso molto improbabile di danno e/o infortunio dovuto alla partecipazione a questo studio, contattare i referenti dello studio filippo.nimbi@uniroma1.it; stefano.eleuteri@uniroma1.it

Riceverò un compenso per la mia partecipazione a questa ricerca?
Non sono previsti compensi o riconoscimenti di alcun tipo per la partecipazione allo studio.

Dove posso andare per fare domande sulla ricerca?
In caso avesse delle domande, osservazioni o reclami in merito allo studio, può mettersi in contatto con i ricercatori:

- Nazionale: filippo.nimbi@uniroma1.it; stefano.eleuteri@uniroma1.it

Per maggiori informazioni sulla protezione dei Suoi dati personali, può contattare il responsabile della protezione dati:

- Nazionale: responsabileprotezionedati@uniroma1.it
-

NB: Aggiungere un disclaimer iniziale dicendo che è uno studio globale, che si ripete in diverse lingue allo stesso modo. Ci scusiamo se alcune domande o risposte potrebbero sembrare fuori contesto o risultare poco rispettose.(Qualcosa del genere, vedi protocollo isc)

1. Selezione			
1.1.	Scelga la Sua lingua	Lista delle lingue dello studio	
1.2.	Vive in Italia?	1 Si<br>2 No	Se 1, vai alla 1.3
1.2.a	Vive in uno dei seguenti paesi?	Lista dei paesi coinvolti nello studio I-SHARE	Se non vive in nessuno di questi paesi, termini il sondaggio. "Sfortunatamente, solo i residenti dei paesi citati nella lista possono partecipare alla ricerca"
1.3	Quanti anni ha?	(numero)	se <18: termini questionario.<br>"sfortunatamente il questionario è solo per persone maggiorenni"<br>Se ha 18 anni, o più, vada al consenso informato

2. Informazioni Sociodemografiche			
2.1.	Quale genere le è stato assegnato alla nascita?	1 Femmina<br>2 Maschio<br>3 Altro, specificare	
2.1.a	Con quale delle seguenti alternative si identifica di più?	1 Maschio<br>2 Femmina<br>3 Entrambi<br>4 Nessuno dei due<br>5 Altro (specificare)	
2.2.	Qual è la miglior descrizione dell'area in cui vive?	1 Capitale<br>2 Città<br>3 Periferia<br>4 Paese/Piccolo centro<br>5 Area rurale/remota<br>6 Altro (specificare)	
2.2. a	PAESE SPECIFICO: in quale regione?	Inserire lista regioni italiane	
2.3.	Qual è la miglior descrizione del suo stato relazionale? *(sono possibili più risposte)*	1 Single e non ho mai avuto un partner<br>2 Single, ma ho avuto un partner in precedenza o mi sto attualmente frequentando con qualcuno<br>3 In una relazione, ma non conviviamo<br>4 Non legalmente sposato/a o unito/a civilmente, ma convivente<br>5 Legalmente sposato/a o unito/a civilmente e convivente<br>6 Legalmente sposato/a o unito/a civilmente, ma non convivente<br>7 Legalmente sposato/a o unito/a civilmente, ma separato/a<br>8 Vedovo/a<br>9 Divorziato/a<br>10 Altro	
2.4.	Quanti figli ha, se ne ha? Risponda 0 se non hai figli	(numero)	
2.5.	Qual è il suo più alto grado di istruzione?	1 Nessuna educazione istituzionale<br>2 Ho frequentato parte della scuola primaria (elementari e medie)	

		3 Ho completato la scuola primaria (elementari e medie)<br><br>4 Ho frequentato parte della scuola secondaria (superiori)<br><br>5 Ho completato la scuola secondaria (superiori)<br><br>6 Ho frequentato parte dell'università o di corsi di specializzazione<br><br>7 Ho completato l'università o corsi di specializzazione<br><br>8 Altro…specificare	
2.6.	Qual è la sua religione?	1 Cristiana Cattolica<br><br>2 Protestante<br><br>3 Ortodossa (Russa/Greca/etc.)<br><br>4 Ebraica<br><br>5 Musulmana<br><br>6 Hindu<br><br>7 Buddhista<br><br>8. Nessuna religione<br><br>9. Altro (specificare)	
2.7.	Qual è la sua origine etnica?	1 Bianca/Caucasica<br><br>2 Asiatica/Oceano Pacifico<br><br>3 Latino-americana/Ispanica<br><br>4 Nera/Africana/Afroamericana<br><br>5 Nativa Americana<br><br>6 Altro specificare	

Introduzione: dal 9 marzo 2020 il Governo ha attuato diverse misure drastiche volte a garantire il distanziamento sociale (lockdown) per contenere la diffusione del COVID-19 in Italia. In questo studio faremo riferimento a queste misure come misure di distanziamento sociale da COVID-19.

3.1.	**Quanto ritiene di seguire/aver seguito le misure di** distanziamento sociale (lockdown) **da COVID-19?**	**1 per niente**<br>**2 poco**<br>**3 molto**<br>**4 In maniera estremamente rigorosa**	
3.2.	È mai stato in (auto)isolamento a causa di sintomi o perché è stato a stretto contatto con qualcuno con il COVID-19 o perché è tornato da un paese che ha avuto un gran numero di casi?	1 No<br>2 Si	
3.3.	Ha mai fatto il test per il COVID-19?	1 No<br>2 Si, sono risultato/a positivo/a almeno una volta<br>3 Si, sono sempre risultato/a negativo/a	
3.4.	Quante persone hanno vissuto in casa sua nei 3 mesi precedenti alle misure di confinamento sociale (lockdown) da COVID-19?<br>Consideri qualsiasi persona che abbia dormito sotto il Suo STESSO tetto per almeno 4 notti a settimana	1 Numero di adulti > 18 anni<br>2 Numero di bambini dai 0 ai 9 anni<br>3 Numero di adolescenti dai 10 ai 18 anni	
3.5.	La sua famiglia era composta diversamente durante le misure di confinamento sociale (lockdown) da COVID-19?	1 No, la composizione della mia famiglia era la stessa<br>2 La composizione della mia famiglia era diversa	Se 1, vai al 3.7.
3.6	Quante persone hanno vissuto in casa sua durante le misure di confinamento sociale (lockdown) da COVID-19?	1 Numero di adulti > 18 anni<br>2 Numero di bambini dai 0 ai 9 anni<br>3 Numero di adolescenti dai 10 ai 18 anni	
3.7.	Quale era il suo lavoro il mese precedente all'attuazione delle misure di confinamento sociale (lockdown) da COVID-19? *(sono possibili più risposte)*	1 Impiegato e retribuito<br>2 Libero professionista/Imprenditore<br>3 Disoccupato<br>4 Lavoro precario/frammentario<br>5 Pensionato<br>6 Studente<br>7 Altro	Se 5, vai al 3.9

3.8	Il suo lavoro ha subito cambiamenti dall'inizio delle misure di confinamento sociale (lockdown) da COVID-19?	1 Nessun cambiamento: ho continuato a fare lo stesso lavoro e mi sono recato/a al solito posto di lavoro<br><br>2 Ho continuato a svolgere lo stesso lavoro, ma da casa (smart working)<br><br>3 Ho continuato a svolgere lo stesso lavoro, ma in parte da casa<br><br>4 Sono impiegato/a e pagato/a, ma impossibilitato/a a lavorare<br><br>5 Lavoro a tempo ridotto<br><br>6 Ho perso il mio lavoro/ impiego/professione<br><br>7 Sono temporaneamente disoccupato/a<br><br>8 Ho cambiato lavoro/impiego<br><br>9 Altro	
3.9.	Di seguito è riportata una scala di reddito in cui 1 indica il gruppo di reddito più basso e 10 il gruppo di reddito più alto nel suo paese. In quale gruppo si trovava la sua famiglia nell'anno precedente alla crisi da COVID-19?<br><br>Si prega di specificare il numero che ritiene più appropriato tenendo conto di tutti i salari, gli stipendi, le pensioni e gli altri redditi.	1 Gruppo di reddito più basso<br><br>2<br><br>3<br><br>4<br><br>5<br><br>6<br><br>7<br><br>8<br><br>9<br><br>10 Gruppo di reddito più alto	
3.10.	Dalla pandemia da COVID-19, la situazione economica di molte famiglie è cambiata. È stato questo il suo caso?	1 Si, la situazione economica della mia famiglia è peggiorata<br><br>2 No, la situazione economica della mia famiglia è rimasta la stessa<br><br>3 Si, la situazione economica della mia famiglia è migliorata	
3.11	Ha avuto una personale esperienza di perdita di reddito?	1 Si, una perdita totale di reddito<br><br>2 Si, una perdita parziale di reddito	

		3 No, nessuna perdita di reddito<br><br>4 Non avevo reddito personale prima del COVID-19	
		Prima delle misure di confinamento sociale (lockdown) da COVID-19	*Durante* le misure di confinamento sociale (lockdown) da COVID-19, ha aumentato o diminuito?
3.12.	Quanto spesso beve alcolici?	1 Mai<br><br>2 Una volta al mese o meno<br><br>3 2-4 volte al mese<br><br>4 2-3 volte a settimana<br><br>5 4 o più volte a settimana	1 Diminuito molto<br><br>2 Diminuito abbastanza<br><br>3 Rimasto invariato<br><br>4 Aumentato abbastanza<br><br>5 Aumentato di molto
3.13.	Di solito, quanti bicchieri di alcolici consuma in una giornata quando beve?	1 1-2<br><br>2 3-4<br><br>3 5-6<br><br>4 7-9<br><br>5 10 +	1 Diminuito molto<br><br>2 Diminuito abbastanza<br><br>3 Rimasto invariato<br><br>4 Aumentato abbastanza<br><br>5 Aumentato di molto
3.14.	Quanto spesso beve 6 o più drink/bicchieri in una sola occasione?	1 Mai<br><br>2 Una volta al mese o meno<br><br>3 2-4 volte al mese<br><br>4 2-3 volte a settimana<br><br>5 4 o più volte a settimana	1 Diminuito molto<br><br>2 Diminuito abbastanza<br><br>3 Rimasto invariato<br><br>4 Aumentato abbastanza<br><br>5 Aumentato di molto
3.15.	Quanto spesso fa uso di droghe leggere (es. hashish, marijuana)?	1 Mai<br><br>2 Una volta al mese o meno<br><br>3 2-4 volte al mese<br><br>4 2-3 volte a settimana<br><br>5 4 o più volte a settimana	1 Diminuito molto<br><br>2 Diminuito abbastanza<br><br>3 Rimasto invariato<br><br>4 Aumentato abbastanza<br><br>5 Aumentato di molto
3.16	Quanto spesso fa uso di altri tipi di droghe (es., cocaina, eroina, droghe sintetiche)?	1 Mai<br><br>2 Una volta al mese o meno<br><br>3 2-4 volte al mese<br><br>4 2-3 volte a settimana<br><br>5 4 o più volte a settimana	1 Diminuito molto<br><br>2 Diminuito abbastanza<br><br>3 Rimasto invariato<br><br>4 Aumentato abbastanza<br><br>5 Aumentato di molto

4. Relazioni familiari e di coppia			
4.1.	Ha avuto un partner stabile nei 3 mesi precedenti le misure di confinamento sociale (lockdown) da COVID-19?	1 No<br>2 Si	Se 1, vai al 4.5.
4.2.	È ancora in questa relazione?	1 No<br>2 Si	Se 2, vai al 4.6
4.3.	La sua relazione è terminata prima, durante o dopo le misure di confinamento sociale (lockdown) da COVID-19?	1 Prima<br>2 Durante<br>3 Dopo	
4.4.	Direbbe che la fine della sua relazione è stata causata dalle misure di confinamento sociale (lockdown) da COVID-19?	1 No<br>2 Si<br>3 Non sono sicuro	
4.5.	Ha un nuovo partener stabile da quando sono in vigore le misure di confinamento sociale (lockdown) da COVID-19?	1 No<br>2 Si	
4.6.	Qual è il suo orientamento sessuale?	1 Asessuale<br>2 Bisessuale<br>3 Gay<br>4 Eterosessuale<br>5 Lesbica<br>6 Pansessuale<br>7 Queer<br>8 Insicuro o incerto<br>9 Altro (specificare)	
4.6. a	Durante le misure di confinamento sociale (lockdown) da COVID-19 il/la suo/a partner stabile ha vissuto con lei nello stesso luogo? *(solo per coloro che hanno risposto 2 alla 4.2 o 2 alla 4.5)*	1 No, lui/lei vive altrove<br>2 Si, per tutto il tempo<br>3 Si, parte del tempo	
4.7.	Nei 3 mesi precedenti all'introduzione delle misure di confinamento sociale (lockdown) da COVID-19, con quale frequenza ha avuto tensioni nella sua relazione con il/la suo/a partner? *(solo per coloro che hanno risposto 2 alla 4.2)*	1 Mai<br>2 Una volta al mese o meno<br>3 2-4 volte al mese<br>4 2-3 volte a settimana<br>5 4 o più volte a settimana	
4.8.	In che modo è cambiata la relazione di coppia da quando sono state introdotte le misure di confinamento sociale (lockdown) da COVID-19? *(solo per coloro che hanno risposto 2 alla 4.2)*	1 Molta meno tensione rispetto a prima<br>2 Un po' meno tensione rispetto a prima<br>3 Circa la stessa quantità di tensione<br>4 Un po' più di tensione rispetto a prima<br>5 Molta più tensione rispetto a prima	

4.9.	Nei 3 mesi precedenti all'introduzione delle misure di confinamento sociale (lockdown) da COVID-19, con quale frequenza ha avuto tensioni nella sua relazione con i suoi figli? *solo per chi vive con bambini (2.4.)*	1 Mai<br>2 Una volta al mese o meno<br>3 2-4 volte al mese<br>4 2-3 volte a settimana<br>5 4 o più volte a settimana		
4.10.	In che modo è cambiata la situazione con i suoi figli da quando sono state introdotte le misure di confinamento sociale (lockdown) da COVID-19? *Solo per chi vive con bambini (2.4.)*	1 Molta meno tensione rispetto a prima<br>2 Meno tensione rispetto a prima<br>3 Circa la stessa quantità di tensione<br>4 Più tensione rispetto a prima<br>5 Molta più tensione rispetto a prima		
4.11.	Nei 3 mesi precedenti all'introduzione delle misure di confinamento sociale (lockdown) da COVID-19, quanto direbbe che il suo partner le ha fornito supporto emotivo? *Solo per coloro che hanno risposto 2 alla 4.2.*	1 Molto supporto<br>2 Abbastanza supporto<br>3 Poco supporto<br>4 Nessun supporto		
4.12.	In che modo è cambiata la situazione da quando sono state introdotte le misure di confinamento sociale (lockdown) da COVID-19? *Solo per chi ha risposto 2 alla 4.2*	1 Molto meno supporto rispetto a prima<br>2 Meno supporto rispetto a prima<br>3 Circa lo stesso supporto rispetto a prima<br>4 Più supporto rispetto a prima<br>5 Molto più supporto rispetto a prima		
4.13.	Chi svolge la maggior parte del lavoro domestico nella sua famiglia? *Solo per coloro che vivono con il/la partner/coniuge*	*Prima* dell'introduzione delle misure di confinamento da COVID-19<br>1 Svolgevo la maggior parte del lavoro domestico<br>2 Il/la mio/a partner svolgeva la maggior parte del lavoro domestico<br>3 Il/la mio/a partner ed io contribuivamo in parti uguali al lavoro domestico<br>4 La maggior parte dei membri della famiglia contribuivano allo stesso modo<br>5 Qualcun altro svolgeva la maggior	*Dopo* l'introduzione delle misure di confinamento da COVID-19<br>1 Svolgevo la maggior parte del lavoro domestico<br>2 Il/la mio/a partner svolgeva la maggior parte del lavoro domestico<br>3 Il/la mio/a partner ed io contribuivamo in parti uguali al lavoro domestico<br>4 La maggior parte dei membri della famiglia contribuivano allo stesso modo<br>5 Qualcun altro svolgeva la maggior parte del lavoro domestico	Solo se le misure sono terminate

		parte del lavoro domestico		
4.14.	Nella sua famiglia chi aveva più controllo delle spese familiari *prima* dell'introduzione delle misure di confinamento sociale (lockdown) da COVID-19? *Solo per coloro che vivono con un/a partner/coniuge (per coloro che hanno risposto 3 o 4 alla 4.6)*	1 Io avevo il maggior controllo<br>2 Il/la mio/a partner aveva il maggior controllo<br>3 Il/la mio/a partner ed io avevamo lo stesso controllo<br>4 Qualcun altro oltre al/alla mio/a partner ed io ha avuto il maggior controllo		
4.15.	Il suo controllo sulle spese familiari è cambiato dall'introduzione delle misure di confinamento sociale (lockdown) da COVID-19? *Solo per chi vive con il partner/coniuge (per coloro che hanno risposto 3 o 4 alla 4.6)*	1 Si, ora esercito il maggior controllo<br>2 Si, ora esercito meno controllo<br>3 No, ora esercito lo stesso controllo		

5. Comportamento sessuale				
5.1.	Ha mai avuto esperienze sessuali?<br><br>Per "esperienza sessuale" intendiamo qualsiasi tipo di esperienza che abbia suscitato un'eccitazione sessuale. Potrebbe trattarsi di baci, contatto fisico, rapporto sessuale, masturbazione, visione di immagini sessualmente esplicite o qualsiasi altra forma di attività sessuale.	1 No<br><br>2 Si		Se 1, vai alla sezione 9
		Nei 3 mesi *precedenti* all'introduzione delle misure di confinamento sociale (lockdown) da COVID-19	*Durante* l'introduzione delle misure di confinamento sociale (lockdown) da COVID-19	
5.2.	Quanto è stata soddisfacente la sua vita sessuale?	1 Molto soddisfacente<br><br>2 Abbastanza soddisfacente<br><br>3 Non molto soddisfacente<br><br>4 Per niente soddisfacente	1 Molto soddisfacente<br><br>2 Abbastanza soddisfacente<br><br>3 Non molto soddisfacente<br><br>4 Per niente soddisfacente	
5.3.	Quanto spesso lei o il suo/a partner avete avuto problemi sessuali (es., problemi ad ottenere l'erezione o perdita di interesse sessuale, eccitazione, orgasmo, soddisfazione sessuale)?<br>*(solo per coloro che hanno risposto 2 alla 4.2 o 2 alla 4.5)*	1 Mai<br><br>2 Una volta<br><br>3 Qualche volta<br><br>4 Spesso<br><br>5 Non applicabile alla mia situazione	1 Mai<br><br>2 Una volta<br><br>3 Qualche volta<br><br>4 Spesso<br><br>5 Non applicabile alla mia situazione	

	Le prossime domande sono volte ad indagare i comportamenti sessuali nei 3 mesi precedenti e durante l'applicazione delle misure di confinamento sociale (lockdown) da COVID-19.	*Nei 3 mesi precedenti* alle misure di confinamento sociale (lockdown) da COVID-19	*Durante* le misure di confinamento sociale (lockdown) da COVID-19
5.4.	Quante volte si è abbracciato/a, baciato/a, tenuto/a per mano o coccolato/a con il suo/a partner stabile? *(per coloro che rispondono 2 alla 4.2 o 2 alla 4.5.)*	1 Mai<br>2 Una volta al mese o meno<br>3 2-4 volte al mese<br>4 2-3 volte a settimana<br>5 4 o più volte a settimana	1 Molto di meno<br>2 Un po' di meno<br>3 Nessuna differenza rispetto a prima<br>4 Un po' di più<br>5 Molto di più
5.5.	Quante volte si è impegnato/a in attività sessuali con il/la suo/a partner stabile? Per attività sessuale si intende un rapporto orale, vaginale, anale o toccarsi *(per coloro che rispondono 2 alla 4.2 o 2 alla 4.5)*	1 Mai<br>2 Una volta al mese o meno<br>3 2-4 volte al mese<br>4 2-3 volte a settimana<br>5 4 o più volte a settimana	1 Molto di meno<br>2 Un po' di meno<br>3 Nessuna differenza rispetto a prima<br>4 Un po' di più<br>5 Molto di più
5.5.a	Quante volte ha utilizzato precauzioni (es., condom, contraccettivi) quando ha fatto sesso con il suo partner stabile?	1 Mai<br>2 Raramente<br>3 Qualche volta<br>4 La maggior parte del tempo<br>5 Sempre *(solo per coloro che hanno risposto 2, 3, 4, 5 alla 5.5)*	1 Molto di meno<br>2 Un pò meno<br>3 Nessuna differenza<br>4 Un pò di più<br>5 Molto di più
5.6.	Quante volte si è masturbato/a?	1 Mai<br>2 Una volta al mese o meno	1 Molto di meno<br>2 Un po' di meno

		3 2-4 volte al mese<br>4 2-3 volte a settimana<br>5 4 o più volte a settimana	3 Nessuna differenza rispetto a prima<br>4 Un po’ di più<br>5 Molto di più
5.7.	Quante volte ha fatto sesso con qualcuno con cui non aveva una relazione a lungo termine (un partner occasionale)?	1 Mai<br>2 Una volta al mese o meno<br>3 2-4 volte al mese<br>4 2-3 volte a settimana<br>5 4 o più volte a settimana	1 Molto di meno<br>2 Un po’ di meno<br>3 Nessuna differenza rispetto a prima<br>4 Un po’ di più<br>5 Molto di più
5.7.a	Quante volte ha utilizzato precauzioni (es., condom, contraccettivi) quando ha fatto sesso con un partner occasionale?	1 Mai<br>2 Raramente<br>3 Qualche volta<br>4 La maggior parte del tempo<br>5 Sempre *(solo per coloro che hanno risposto 2, 3, 4, 5 alla 5.7)*	1 Molto di meno<br>2 Un pò meno<br>3 Nessuna differenza<br>4 Un pò di più<br>5 Molto di più
5.8.	Quante volte ha inviato o ricevuto foto, video di nudo/semi-nudo (sexting) o audio/vocali a contenuto erotico?	1 Mai<br>2 Una volta al mese o meno<br>3 2-4 volte al mese<br>4 2-3 volte a settimana<br>5 4 o più volte a settimana	1 Molto di meno<br>2 Un po’ di meno<br>3 Nessuna differenza rispetto a prima<br>4 Un po’ di più<br>5 Molto di più
5.9.	Quante volte ha fatto sesso in cambio di denaro, beni materiali, favori, droghe o rifugio?<br><br>Per beni materiali intendiamo cose come cibo, affitto, vestiti/scarpe/telefoni cellulari, cosmetici, trasporti, buoni voti a scuola o tasse scolastiche o articoli per i suoi figli, la sua famiglia o se stesso/a	1 Mai<br>2 Una volta al mese o meno<br>3 2-4 volte al mese<br>4 2-3 volte a settimana<br>5 4 o più volte a settimana	1 Molto di meno<br>2 Un po’ di meno<br>3 Nessuna differenza rispetto a prima<br>4 Un po’ di più<br>5 Molto di più
5.10.	Quante volte ha guardato video sessualmente espliciti (pornografia)?	1 Mai<br>2 Una volta al mese o meno<br>3 2-4 volte al mese<br>4 2-3 volte a settimana<br>5 4 o più volte a settimana	1 1 Molto di meno<br>2 Un po’ di meno<br>3 Nessuna differenza rispetto a prima<br>4 Un po’ di più<br>5 Molto di più
5.11.	Quante volte ha messo in atto/guardato atti sessuali tramite webcam?	1 Mai<br>2 Una volta al mese o meno<br>3 2-4 volte al mese<br>4 2-3 volte a settimana<br>5 4 o più volte a settimana	1 Molto di meno<br>2 Un po’ di meno<br>3 Nessuna differenza rispetto a prima<br>4 Un po’ di più<br>5 Molto di più
5.12	Se alcuni dei suoi comportamenti sessuali sono cambiati a causa dell’introduzione delle misure di confinamento sociale (lockdown) da COVID-19, quale potrebbe essere il motivo?	Risposta aperta	
	Accesso ai preservativi		
5.13	Le misure di confinamento sociale (lockdown) da COVID-19 hanno reso più difficile l'accesso ai preservativi? (non per chi ha risposto 1 alla 5.1)	1 No<br><br>2 Si<br><br>3 Non applicabile – Normalmente non faccio uso di preservativi	Se 1 o 3, vai alla sezione 6 (donne) o 9 (uomini)
5.14	Se sì, cosa ha reso difficile l’accesso ai preservativi? *(non per chi ha risposto 1 alla 5.1)*	1 Nessun trasporto disponibile<br><br>2 Paura di poter contrarre il COVID-19 e quindi non volevo/voglio andare dal medico/centro sanitario/negozio<br><br>3 I negozi erano/sono chiusi<br><br>4 I preservativi non erano/sono disponibili nel mio negozio di fiducia	Tutti gli uomini, vai alla sezione 9

		5 Non ero/sono in grado o non avevo/ho il permesso di uscire di casa<br><br>6 Farmacia e distributori chiusi o fuori servizio<br><br>7 La struttura clinica aveva/ha lunghe code o non era/è accessibile<br><br>8 Non potevo/posso più permettermi di comprarli<br><br>9 Non riuscivo/riesco più ad accedere ai preservativi gratuiti<br><br>10 Altro…	

6. Accesso ai contraccettivi			
6.1.	É mai rimasta incinta?	1 No<br>2 Si	Se 1, vai alla 6.3
6.2.	Quante volte è rimasta incinta nella sua vita?	(numero)	
6.3.	Quale frase descrive meglio la sua situazione attuale?	1 Sono attualmente o probabilmente incinta<br>2 Attualmente sto cercando di rimanere incinta<br>3 Ho avuto di recente un bambino (durante o successivamente le misure di confinamento sociale da COVID-19)<br>4 Attualmente non sono incinta e non desidero esserlo in un futuro prossimo<br>5 Non posso avere figli (problemi di fertilità/problemi di natura medica/menopausa)	Se 1, vai alla sezione 7.<br>Se 2, vai alla sezione 9.<br>Se 3, vai alla 7.9.<br>Se 4, continua<br>Se 5, vai alla sezione 9-.
6.4.	Ha recentemente cambiato idea riguardo l'avere un figlio a causa del COVID-19? (solo per coloro che hanno risposto 2 o 4 alla 6.3)	1 Si, ho deciso di rimandare la decisione di avere un figlio ad un futuro prossimo<br>2 Si, ho deciso di avere un figlio il prima possibile<br>3 No, non ho cambiato i miei piani	
6.5.	Lei o il suo partner state facendo qualcosa al fine di evitare o ritardare una gravidanza, incluso l'uso di preservativi, metodi contraccettivi, metodi tradizionali	1 No<br>2 Si, sempre<br>3 Si, la maggior parte delle volte<br>4 Si, qualche volta	Se 2, 3 o 4 vai alla 6.7.
6.6.	Qual è il motivo principale per cui non sta facendo uso di contraccezione?	1 Non sono sessualmente attiva con regolarità e non ho bisogno di contraccettivi<br>2 Non so quale sia il metodo migliore da usare<br>3 Sono spaventata dagli effetti collaterali<br>4 Il/la mio/a partner non vuole<br>5 Altro	Continua alla sezione 8.
6.7.	Al momento, quale metodo contraccettivo sta usando? *(possibili risposte multiple)*	1 Preservativo maschile/femminile<br>2 Diaframma<br>3 Pillola<br>4 Cerotto/anello<br>5 Spirale (IUD) a base di rame<br>6 Spirale (IUD) a base ormonale	

		7 Impianto contraccettivo ormonale sottocutaneo<br><br>8 Contraccettivi per via iniettiva<br><br>9 Sterilizzazione mia o del partner<br><br>10 Coito interrotto<br><br>11 Metodi naturali (es., Ogino-Knaus, metodo Billings)<br><br>12 App per il controllo delle nascite<br><br>13 Altro…	
6.8.	Le misure di confinamento sociale (lockdown) da COVID-19 l'hanno *fermata o ostacolata* dal cercare o ottenere dei contraccettivi?	1 No<br><br>2 Si	Se no, vai alla 6.10
6.9.	Cosa l'ha fermata o ostacolata dal cercare o ottenere dei contraccettivi? *(sono possibili più risposte)*	1 Nessun trasporto disponibile<br><br>2 Paura di poter contrarre il COVID-19 e quindi non colevo/voglio andare dal medico/centro sanitario/negozio<br><br>3 Non ero/sono in grado o non avevo/ho il permesso di uscire di casa<br><br>4 Metodo non disponibile<br><br>5 Medico/professionista sanitario non disponibile<br><br>6 Farmacia e distributore chiusi o fuori servizio<br><br>7 Non potevo/posso più permettermi di comprarlo<br><br>8 La struttura clinica aveva/ha lunghe code o non era/è accessibile<br><br>9 Altro	
6.10.	Quali servizi utilizzava per cercare o ottenere contraccettivi *prima* delle misure di confinamento sociale (lockdown) da COVID-19? *(più risposte possibili)*	1 Medico di famiglia/ Medico generico<br><br>2 Medico/infermiere ospedaliero<br><br>3 Centri di salute pubblici (ASL, consultori)<br><br>4 Servizi on-line<br><br>5 Servizi telefonici<br><br>6 Servizi da banco (farmacia)<br><br>7 Altro	
6.11.	Quali servizi ha usato per cercare o ottenere contraccettivi *durante* il periodo in cui erano in atto le misure di confinamento sociale (lockdown) da COVID-19? (sono possibili più risposte)?	1 Medico di famiglia/ Medico generico<br><br>2 Medico/infermiere ospedaliero<br><br>3 Centri di salute pubblici (ASL, consultori)<br><br>4 Servizi on-line<br><br>5 Servizi telefonici	Se non hai risposto sì alla 6.3, vai alla sezione 8

		6 Servizi da banco (farmacia)<br><br>7 Altro<br><br>8 Non avevo bisogno di cercare o ottenere contraccettivi durante le misure di confinamento sociale (lockdown) da COVID-19	

7. Accesso ai servizi di salute riproduttiva, assistenza prenatale, gravidanza e salute materna e infantile (solo le donne che rispondono sì alla 6.3.)			
7.1.	Da quanti mesi è incinta?	1-9	
7.1.a	Quando ha scoperto di essere incinta, qual è stata la sua reazione?	1 Molto triste<br>2 Abbastanza triste<br>3 Piuttosto felice<br>4 Molto felice	
7.1.b	Aveva programmato di rimanere incinta?	1 Si<br>2 Si, ma è successo troppo presto rispetto a quando l'avevamo programmato<br>3 Si, ma è successo più tardi rispetto a quando l'avevamo programmato<br>4 No	
7.1.c.	Secondo lei, il fatto di essere rimasta incinta è collegato alla situazione causata dal COVID-19?	1 No<br>2 Si, non ho avuto accesso ai contraccettivi a causa del COVID-19<br>3 Si, non ho avuto accesso ai contraccettivi d'emergenza (es., pillola del giorno dopo) a causa del COVID-19<br>4 Si, ho avuto bisogno di soldi/regali da una relazione sessuale e sono rimasta incinta<br>5 Si, c'era più tempo libero a causa della chiusura di scuole e aziende e ho potuto avere più attività sessuale<br>6 Si, ci sono più violenze e abusi sessuali nel mio paese in questo periodo e sono rimasta incinta a seguito di una violenza<br>7 Altro (specificare)	
7.1.d.	Hai deciso di portare avanti la gravidanza?	1 Si<br>2 No, ho deciso di terminare la gravidanza<br>3 Non lo so ancora	Se 2 vai alla 8.
7.2.	Ha perso o ritardato gli appuntamenti di assistenza sanitaria in gravidanza durante le misure di confinamento sociale (lockdown) da COVID-19?<br>(Alcuni operatori hanno visto i loro pazienti per telefono o tramite videoconferenza. NON contiamo questi tipi di visite come perse.)	1 No<br>2 Si, perché temevo di poter contrarre il COVID-19 nel centro ospedaliero/sanitario<br>3 Sì, perché il medico/l'infermiere ha annullato o riprogrammato l'appuntamento a causa del COVID-19<br>4 Si, per altri motivi	
7.3.	Quanto è soddisfatta della sua assistenza sanitaria in gravidanza durante le misure di confinamento sociale (lockdown) da COVID-19?	1 Per nulla soddisfatta<br>2 Non soddisfatta	

		3 Né soddisfatta, né insoddisfatta<br>4 Un po' soddisfatta<br>5 Molto soddisfatta	
7.4.	A causa del COVID-19, si è sentita ansiosa o depressa durante la gravidanza?	1 No<br>2 Si, un po'<br>3 Si, molto	
7.5.	Ha ricevuto informazioni riguardo la trasmissione del COVID-19 durante la gravidanza?	1 No<br>2 Sì, dal mio medico/ostetrica<br>3 Si, dai media<br>4 Si, da altre fonti	
7.6.	Ha avuto dubbi rispetto al parto nelle settimane/mesi seguenti?	1 No<br>2 Sì, temevo di poter contrarre il COVID-19 nel centro ospedaliero/sanitario<br>3 Sì, temevo di non sapere come arrivare in ospedale<br>4 Si, per altri motivi	
7.7.	Dove aveva programmato di far nascere il bambino?	1 Al centro sanitario o in ospedale<br>2 A casa con un operatore sanitario<br>3 A casa con una balia o altra figura non sanitaria<br>4 A casa da sola<br>5 Altro…	Se 1 o 5 vai alla sezione 9.
7.8.	Perché ha deciso di partorire a casa?	1 Ero preoccupata per il rischio di contrarre il COVD-19 nelle strutture sanitarie<br>2 La struttura era chiusa o non poteva fornire assistenza<br>3 Non ho avuto accesso alla struttura<br>4 Preferivo partorire a casa	Vai alla sezione 9
7.9.	Dove ha partorito?	1 Al centro sanitario o in ospedale<br>2 A casa con un operatore sanitario<br>3 A casa da sola<br>4 A casa con una balia o altra figura non sanitaria<br>5 Altro	Se 1 o 5, vai alla 7.11
7.10.	Perché ha partorito a casa?	1 Ero preoccupata per il rischio di contrarre il COVID-19 nelle strutture sanitarie<br>2 La struttura era chiusa o non poteva fornire assistenza<br>3 Non avevo accesso ad una struttura<br>4 Preferivo partorire a casa	

7.11.	Ha perso o ritardato gli appuntamenti di assistenza postnatale a seguito delle misure attuate a causa del COVID-19?<br><br>(Alcuni operatori hanno visto i loro pazienti per telefono o tramite videoconferenza. NON contiamo questi tipi di visite come perse.)	1 No<br><br>2 Sì, perché avevo paura di andare ai servizi sanitari<br><br>3 Sì, perché il medico/l'infermiere ha annullato o riprogrammato l'appuntamento<br><br>4 Si, per altri motivi	

8. Aborto (solo donne)			
8.1.	Durante le misure di confinamento sociale (lockdown) da COVID-19 ha interrotto una gravidanza (abortire)?	1 No<br><br>2 Si	se 1, vai alla sezione 9.
8.2.	Ha avuto un aborto durante le misure di confinamento sociale (lockdown) da COVID-19?	1 No<br><br>2 Si, un aborto medico (assunzione di pillole, e.g. misoprostol, o erbe)<br><br>3 Sì, un aborto chirurgico<br><br>4 Si, con altri metodi<br><br>5 Si, spontaneo	
8.3.	La situazione dovuta al COVID-19 le ha ***impedito*** di ottenere un'interruzione volontaria di gravidanza (aborto)?	1 No<br><br>2 Si	Se 1 alla 8.2 e 1 alla 8.3., vai alla sezione 9
8.4.	In che modo le misure di distanziamento sociale da COVID-19 le hanno impedito di ottenere un'interruzione volontaria di gravidanza (aborto)?	1 Nessun trasporto disponibile<br><br>2 Paura di contrarre il COVID-19 e quindi non volevo andare dal medico/centro sanitario per abortire<br><br>3 Non ero in grado o non avevo il permesso di uscire di casa<br><br>4 Servizio per interruzione di gravidanza non disponibile<br><br>5 Medico/professionista sanitario non disponibile<br><br>6 Farmacia/distributore chiusi o fuori servizio<br><br>7 Non potevo più permettermi economicamente un'interruzione di gravidanza<br><br>8 Il centro sanitario/clinica aveva lunghe code o non era accessibile<br><br>9 Altro	
8.5.	Quali servizi avrebbe usato per un'interruzione volontaria di gravidanza (aborto) ***prima*** delle misure di confinamento sociale (lockdown) da COVID-19? (sono possibili più risposte)?	1 Non ho mai avuto un aborto prima delle misure di confinamento sociale (lockdown) da COVID-19<br><br>2 Medico di famiglia/Medico generico<br><br>3 Medico/infermiere ospedaliero<br><br>4 Servizi on-line<br><br>5 Servizi telefonici<br><br>6 Servizi da banco (farmacia)<br><br>7 Guaritore tradizionale<br><br>8 Automedicazione<br><br>9 Clinica per aborto<br><br>10 Attraverso un'associazione sociale che si occupa di aborto<br><br>11 Altro	

8.6.	Quali servizi ha usato per ottenere un'interruzione volontaria di gravidanza (aborto) ***durante*** le misure di confinamento sociale (lockdown) da COVID-19? (sono possibili più risposte)?	1 Medico di famiglia/Medico generico<br>2 Medico/infermiere ospedaliero<br>3 Servizi on-line<br>4 Servizi telefonici<br>5 Servizi da banco (farmacia)<br>6 Guaritore tradizionale<br>7 Automedicazione<br>8 Clinica per aborto<br>9 Attraverso un'associazione sociale che si occupa di aborto<br>10 Altro	
8.7.	Ha avuto qualche ritardo nell'ottenere assistenza per un'interruzione volontaria di gravidanza (aborto)?	1 No<br>2 Si, un po' di giorni<br>3 Si, 1-2 settimane<br>4 Si, 3-4 settimane<br>5 Si, più di 4 settimane	

9. Violenza sessuale e di genere				
9.1	Nella sua vita quotidiana, nei 3 mesi precedenti il COVID-19, quanto si è sentito/a vulnerabile a molestie sessuali o aggressioni sessuali, fisiche o emotive, da parte di qualcuno che non vive in casa sua?	1 Non vulnerabile<br>2 Leggermente vulnerabile<br>3 Neutrale<br>4 Un po' vulnerabile<br>5 Molto vulnerabile		
9.2.	Nella sua vita quotidiana, durante la pandemia di COVID-19, quanto si è sentito/a vulnerabile a molestie sessuali o aggressioni sessuali, fisiche o emotive, da parte di qualcuno che non vive in casa sua?	1 Non vulnerabile<br>2 Leggermente vulnerabile<br>3 Neutrale<br>4 Un po' vulnerabile<br>5 Molto vulnerabile		
		Nei 3 mesi precedenti alle misure di confinamento sociale (lockdown) da COVID-19	*Durante* le misure di confinamento sociale (lockdown) da COVID-19	
9.1.	Un/una partner ha cercato di limitare/impedire il suo contatto (on-line o telefonico) con la sua famiglia?	1 No<br>2 Si, una volta<br>3 Si, più volte<br>4 Non applicabile alla mia situazione	1 No<br>2 Si, una volta<br>3 Si, più volte<br>4 Non applicabile alla mia situazione	
9.2.	Un/a partner l'ha insultato/a o fatta stare/sentire male?	1 No<br>2 Si, una volta<br>3 Si, più volte<br>4 Non applicabile alla mia situazione	1 No<br>2 Si, una volta<br>3 Si, più volte<br>4 Non applicabile alla mia situazione	
9.3.	Pur avendo denaro a disposizione, le è capitato che il/la suo/a partner non le abbia fornito i soldi per la gestione della casa o dei bambini?	1 No<br>2 Si, una volta<br>3 Si, più volte<br>4 Non applicabile alla mia situazione	1 No<br>2 Si, una volta<br>3 Si, più volte<br>4 Non applicabile alla mia situazione	
9.4.	Un/a partner l'ha schiaffeggiato/a, spinto/a, colpito/a, preso/a a calci o soffocato/a o le ha lanciato qualcosa che avrebbe potuto farle del male?	1 No<br>2 Si, una volta<br>3 Si, più volte<br>4 Non applicabile alla mia situazione	1 No<br>2 Si, una volta<br>3 Si, più volte<br>4 Non applicabile alla mia situazione	
9.5.	Un/a partner l'ha fisicamente costretto/a ad avere rapporti sessuali quando non voleva?	1 No<br>2 Si, una volta<br>3 Si, più volte	1 No<br>2 Si, una volta<br>3 Si, più volte	

		4 Non applicabile alla mia situazione	4 Non applicabile alla mia situazione	
9.6.	Si è mai sentita/o costretta/o ad avere rapporti sessuali quando non aveva voglia perché aveva paura di ciò che il/la suo/a partner avrebbe potuto fare?	1 No<br>2 Si, una volta<br>3 Si, più volte<br>4 Non applicabile alla mia situazione	1 No<br>2 Si, una volta<br>3 Si, più volte<br>4 Non applicabile alla mia situazione	

9.7.	In caso di risposta affermativa a una qualsiasi delle precedenti domande sul COVID-19:<br>Ha mai parlato con qualcuno delle esperienze di violenza che ha avuto *prima* delle misure di confinamento sociale (lockdown) da COVID-19? ***(sono possibili più risposte)***	1 No<br>2 Si, a un parente<br>3 Si, a un amico<br>4 Sì, a una linea di assistenza telefonica o online<br>5 Si, ai servizi sociali<br>6 Si, alla polizia o alle forze dell'ordine<br>7 Si, ad un'associazione<br>8 Si, altro…	
9.8.	Ha mai riportato ufficialmente (ad esempio tramite una denuncia) eventuali esperienze di violenza che ha avuto *prima* delle misure di confinamento sociale (lockdown) da COVID-19?	1 No<br>2 Si	
9.9.	In caso di risposta affermativa su una qualsiasi delle domande sul COVID-19 in corso:<br>Ha mai parlato con qualcuno delle esperienze di violenza che ha avuto *durante* le misure di confinamento sociale (lockdown) da COVID-19? ***(sono possibili più risposte)***	1 No<br>2 Si, a un parente<br>3 Si, a un amico<br>4 Sì, a una linea di assistenza telefonica o online<br>5 Si, ai servizi sociali<br>6 Si, alla polizia o alle forze dell'ordine<br>7 Si, ad un'associazione<br>8 Si, altro…	
9.10.	Ha mai riportato ufficialmente (ad esempio tramite una denuncia) eventuali esperienze di violenza che ha avuto *durante* le misure di confinamento sociale (lockdown) da COVID-19?	1 No<br>2 Si	

10. Opzionale: mutilazione genitale femminile e matrimonio combinato in età giovanile			
10.1.	Il matrimonio combinato in età giovanile (prima dei 18 anni) avviene nella sua comunità?	1 No<br>2 Si<br>3 Non lo so	Se 1 o 3, vai alla 10.6
10.2.	Ha figli con età dai 10 ai 18 anni?	1 No<br>2 Si, uno<br>3 Si, più di uno	Se no, vai alla 10.5
10.3.	*Prima* delle misure di confinamento sociale (lockdown) da COVID-19, aveva intenzione di organizzare un matrimonio combinato per suo figlio/a di età compresa tra 10 e 18 anni?	1 No<br>2 Si	
10.4.	La situazione dovuta al COVID-19 ha cambiato i suoi piani per organizzare un matrimonio combinato per suo figlio/a/i adolescente/i? ***(sono possibili più risposte)***	1 No<br>2 Sì, organizzerò il/i matrimonio/i prima del previsto<br>3 Sì, organizzerò il/i matrimonio/i più tardi del previsto<br>4 Si, ho annullato i piani per il matrimonio	
10.5.	In generale, ritiene che a causa del COVID-19, ragazze e ragazzi abbiano un rischio maggiore di avere un matrimonio combinato in giovane età?	1 No<br>2 Sì, un rischio leggermente più alto<br>3 Si, un rischio molto più alto<br>4 Non lo so	
10.6.	La circoncisione/infibulazione o altra pratica di mutilazione genitale femminile è esercitata nella sua comunità?	1 No<br>2 Si<br>3 Non lo so	Se 1 o 3 vai alla sezione 11
10.7.	Ha una figlia che è nell'età in cui generalmente si pratica la circoncisione/infibulazione?	1 No<br>2 Si	Se no, vai alla 10.10
10.8.	*Prima* delle misure di confinamento sociale (lockdown) da COVID-19, aveva intenzione di circoncidere/infibulare sua figlia?	1 No<br>2 Si	
10.9.	La situazione dovuta al COVID-19 ha cambiato i Suoi piani di circoncidere/infibulare sua figlia?	1 No<br>2 Sì, ho deciso di farle fare la circoncisione/infibulazione prima del previsto<br>3 Sì, ho deciso di farle fare la circoncisione/infibulazione più tardi del previsto<br>4 Sì, ho annullato i piani di circoncisione/infibulazione	
10.10.	In generale, ritiene che a causa del COVID-19, le ragazze abbiano un rischio maggiore di circoncisione/infibulazione?	1 No<br>2 Sì, un rischio leggermente più elevato<br>3 Si, un rischio molto più alto	

		4 Non lo so	

<table>
<tr><th colspan="5">11. HIV/IST</th></tr>
<tr><td>11.1.</td><td>Durante le misure di confinamento sociale (lockdown) da COVID-19, ha desiderato effettuare un test per l'HIV o per un'altra infezione a trasmissione sessuale?</td><td colspan="2">1 No
2 Si</td><td>Se 1, vai alla 11.5.</td></tr>
<tr><td>11.2.</td><td>La situazione dovuta la COVID-19 l'ha ostacolata o le ha impedito di accedere a un test per l'HIV o per un'altra infezione a trasmissione sessuale?</td><td colspan="2">1 No
2 Si</td><td>Se 1, vai alla 11.4.</td></tr>
<tr><td>11.3.</td><td>In che modo le misure di confinamento sociale (lockdown) da COVID-19 l'hanno ostacolata o le hanno impedito di accedere a un test per l'HIV o per un'altra infezione a trasmissione sessuale?</td><td colspan="2">1 Nessun trasporto disponibile
2 I servizi postali non funzionavano
3 Farmacia chiusa
4 Non potevo permettermelo
5 Il centro sanitario/clinica aveva lunghe code o non era accessibile
6 Non ero in grado o non avevo il permesso di uscire
7 Gli operatori sanitari non offrivano più servizi di test per HIV/ IST
8 Altro…</td><td></td></tr>
<tr><td rowspan="2">11.4.</td><td rowspan="2">Quali servizi ha usato per ottenere un test per l'HIV o per un'altra infezione a trasmissione sessuale? (sono possibili più risposte)?</td><td>Prima delle misure di confinamento sociale (lockdown) da COVID-19</td><td>Durante le misure di confinamento sociale (lockdown) da COVID-19</td><td></td></tr>
<tr><td>0 Non ho mai avuto bisogno di un test prima del COVID-19
1 Medico di famiglia/ Medico generico
2 Ospedale/clinica
3 Clinica per HIV/IST
4 Servizi on-line
5 Servizi telefonici
6 Servizi da banco (farmacia)
7 Cure tradizionali
8 Automedicazione
9 Niente
10 Altro</td><td>1 Medico di famiglia/ Medico generico
2 Ospedale/clinica
3 Clinica per HIV/IST
4 Servizi on-line
5 Servizi telefonici
6 Servizi da banco (farmacia)
7 Cure tradizionali
8 Automedicazione
9 Niente
10 Altro</td><td></td></tr>
<tr><td>11.5.</td><td>Nella sua vita, è mai risultato/a positivo/a all'HIV?</td><td colspan="2">1 No
2 Si
3 Non mi va di rispondere</td><td>Se 1 o 3, vai alla sezione 12</td></tr>
</table>

11.6.	*Durante* le misure di confinamento sociale (lockdown) da COVID-19, le è stato annullato un qualsiasi appuntamento presso il suo centro sanitario per il trattamento dell'HIV?	1 No<br><br>2 Si	
11.7.	*Durante* le misure di confinamento sociale (lockdown) da COVID-19, ha perso o posticipato un appuntamento presso il suo centro sanitario per il trattamento dell'HIV?	1 No<br><br>2 Si	Se 1, vai alla 11.9.
11.8.	Qual è stato il motivo principale per cui ha perso o ritardato un appuntamento presso il suo centro sanitario per il trattamento dell'HIV?	1 Nessun trasporto disponibile<br><br>2 Paura di contrarre il COVID-19 se fossi andato/a presso il mio centro sanitario per il trattamento dell'HIV<br><br>3 Non sono in grado/non ho il permesso di uscire di casa<br><br>4 Medico/professionista sanitario non disponibile<br><br>5 Farmacia/distributore chiusi o fuori uso<br><br>6 Non posso più permettermelo<br><br>7 Il centro sanitario/clinica ha lunghe file o al momento non è accessibile<br><br>8 Altro	
11.9.	In che modo le misure di confinamento sociale (lockdown) da COVID-19 hanno influenzato la sua aderenza ai farmaci per l'HIV (su una scala da 1 a 5)?	1 Ha reso impossibile sottoporsi alla terapia antiretrovirale<br><br>2 Ha reso più difficile sottoporsi alle cure<br><br>3 Non ha influito sul sottoporsi alla terapia antiretrovirale<br><br>4 ha reso il sottoporsi alle cure in qualche modo più semplice<br><br>5 Ha reso molto più facile il sottoporsi alla terapia antiretrovirale	
11.10.	Durante le misure di confinamento sociale (lockdown) da COVID-19, è stato preoccupato di rimanere senza farmaci antiretrovirali per l'HIV a causa del lockdown?	1 Molto preoccupato/a<br><br>2 Un po' preoccupato/a<br><br>3 Per nulla preoccupato/a	
11.11.	Le misure di confinamento sociale (lockdown) da COVID-19 l'hanno spinta a rivelare a qualcuno il suo stato di sieropositività?	0 no, continuo a tenerlo privato<br><br>1 no, lo avevo già rivelato<br><br>2 si, mi hanno costretto/a a rivelarlo<br><br>3 sì, anche se avevo intenzione di rivelarlo comunque	

12. Opzionale: salute mentale			
	Fin dalla scorsa settimana:		
12.1.	Mi arrabbio spesso alla minima provocazione.	Completamente d'accordo<br>D'accordo<br>Né in accordo né in disaccordo<br>In disaccordo<br>Completamente in disaccordo	
12.2.	Ciò accade di più o di meno dall'inizio delle misure di contenimento sociale da COVID-19?	Molto di più<br>Di più<br>Più o meno lo stesso<br>Di meno<br>Molto meno	
12.3.	Mi sono sentito/a frustrato/a per le cose in generale.	Completamente d'accordo<br>D'accordo<br>Né in accordo né in disaccordo<br>In disaccordo<br>Completamente in disaccordo	
12.4.	Ciò accade di più o di meno dall'inizio delle misure di contenimento sociale da COVID-19?	Molto di più<br>Di più<br>Più o meno lo stesso<br>Di meno<br>Molto di meno	
12.5.	Mi sono sentito/a annoiato/a.	Completamente d'accordo<br>D'accordo<br>Né in accordo né in disaccordo<br>In disaccordo<br>Completamente in disaccordo	
12.6.	Ciò accade di più o di meno dall'inizio delle misure di contenimento sociale da COVID-19?	Molto di più<br>Di più<br>Più o meno lo stesso<br>Di meno<br>Molto di meno	
12.7.	Sono preoccupato/a per la mia situazione finanziaria.	Completamente d'accordo<br>D'accordo<br>Né in accordo né in disaccordo	

		In disaccordo<br>Completamente in disaccordo	
12.8.	Ciò accade di più o di meno dall'inizio delle misure di contenimento sociale da COVID-19?	Molto di più<br>Di più<br>Più o meno lo stesso<br>Di meno<br>Molto di meno	
	Generali:		
12.9.	Mi sento frustrato/a a causa delle restrizioni da COVID-19	Molto di più<br>Di più<br>Più o meno lo stesso<br>Di meno<br>Molto di meno	
12.10.	Sono confuso/a su ciò che posso o non posso fare a causa del COVID-19.	Completamente d'accordo<br>D'accordo<br>Né in accordo né in disaccordo<br>In disaccordo<br>Completamente in disaccordo	
12.11.	Ho paura di contrarre il COVID-19	Molto di più<br>Di più<br>Più o meno lo stesso<br>Di meno<br>Molto di meno	
12.12.	Metto in atto comportamenti ossessivi o compulsivi riguardo al lavaggio delle mani.	Completamente d'accordo<br>D'accordo<br>Né in accordo né in disaccordo<br>In disaccordo<br>Completamente in disaccordo	
12.13.	Ho paura di toccare oggetti fuori di casa.	Molto di più<br>Di più<br>Più o meno lo stesso<br>Di meno<br>Molto di meno	
12.14.	Non riesco a smettere di pensare all'epidemia di COVID-19.	Completamente d'accordo<br>D'accordo	

		Né in accordo né in disaccordo<br>In disaccordo<br>Completamente in disaccordo	
12.15.	Ho degli incubi sulla situazione attuale.	Completamente d'accordo<br>D'accordo<br>Né in accordo né in disaccordo<br>In disaccordo<br>Completamente in disaccordo	
12.16	Sento che c'è abbastanza equipaggiamento protettivo disponibile per me (guanti, mascherine per la bocca, alcool sterilizzante).	Completamente d'accordo<br>D'accordo<br>Né in accordo né in disaccordo<br>In disaccordo<br>Completamente in disaccordo	
12.17.	Ritengo che il Governo non sia in grado di fornire informazioni sufficienti, adeguate e veritiere sull'epidemia da COVID-19.	Completamente d'accordo<br>D'accordo<br>Né in accordo né in disaccordo<br>In disaccordo<br>Completamente in disaccordo	
12.18.	Se devo starnutire o tossire in casa, cerco di nasconderlo alle persone che mi circondano.	Completamente d'accordo<br>D'accordo<br>Né in accordo né in disaccordo<br>In disaccordo<br>Completamente in disaccordo	
12.19.	Se sono fuori e devo starnutire o tossire, proverei a nasconderlo alle persone intorno a me	Completamente d'accordo<br>D'accordo<br>Né in accordo né in disaccordo<br>In disaccordo<br>Completamente in disaccordo	
12.20.	Come giudica la sua salute mentale generale?	Scarsa<br>Accettabile<br>Buona<br>Molto buona<br>Eccellente	
Nelle ultime due settimane, quante volte è stato/a disturbato/a da ...			

12.21.	Nelle ultime due settimane, quante volte è stato/a disturbato/a da sentimenti di tristezza, calo dell’umore o mancanza di speranza?	Completamente d’accordo<br>D’accordo<br>Né in accordo né in disaccordo<br>In disaccordo<br>Completamente in disaccordo	1 Mai<br>2 Raramente<br>3 Qualche volta<br>4 La maggior parte del tempo<br>5 Sempre
12.22.	Ciò accade di più o di meno dall'inizio del lockdown?	Molto di più<br>Di più<br>Più o meno lo stesso<br>Di meno<br>Molto di meno	

13. Opzionale: Nutrizione			
13.1.	Durante le misure attuate per il COVID-19, temeva che la sua famiglia sarebbe rimasta senza cibo a sufficienza?	1 No<br>2 Si, ma meno rispetto a prima del lockdown<br>3 Si, uguali a prima del lockdown<br>4 Si, più di prima del lockdown	
13.2.	Durante le misure attuate per il COVID-19, lei o qualche membro della famiglia siete stati limitati nel mangiare i vostri cibi preferiti a causa della mancanza di risorse (es, non erano disponibili al supermercato)?	1 No<br>2 Si, ma meno rispetto a prima del lockdown<br>3 Si, uguali a prima del lockdown<br>4 Si, più di prima del lockdown	
13.3.	Durante le misure attuate per il COVID-19, lei o qualsiasi membro della famiglia avete mangiato meno durante i pasti perché non c'era abbastanza cibo?	1 No<br>2 Si, ma meno rispetto a prima del lockdown<br>3 Si, uguali a prima del lockdown<br>4 Si, più di prima del lockdown	
13.4.	Durante le misure attuate per il COVID-19, i negozi di alimentari vicino a Lei erano mai completamente vuoti e senza possibilità di rifornirli?	1 No<br>2 Si, ma meno rispetto a prima del lockdown<br>3 Si, uguali a prima del lockdown<br>4 Si, più di prima del lockdown	
13.5.	Durante le misure attuate per il COVID-19, ha aumentato il consumo di alimenti a basso valore nutrizionale (es., fast food, merendine, ecc.)?	1 No<br>2 Si, un po'<br>3 Si, molto	
13.6.	Durante le misure attuate per il COVID-19, ha aumentato il consumo di cibo in generale?	1 No<br>2 Si, un po'<br>3 Si, molto	

Grazie per la Sua partecipazione! Qui può trovare una lista di associazioni [FARE UNA LISTA DI ASSOCIAZIONI LOCALI] che può contattare in caso dovesse avere bisogno di aiuto. È inoltre possibile scaricare queste informazioni per un riferimento futuro.
